# Research Progress on the Physiological Functions of Aspartic Acid and Its Applications in Animal Production

**DOI:** 10.3390/ani16071016

**Published:** 2026-03-26

**Authors:** Xiwen Zhang, Qi Luo, Yurong Zhao

**Affiliations:** College of Animal Science and Technology, Hunan Agricultural University, Changsha 410128, China; zxw18985450689@stu.hunau.edu.cn (X.Z.);

**Keywords:** aspartic acid, metabolism, aspartate transporter, biologic activity, animal husbandry

## Abstract

Aspartic acid, a non-essential amino acid required for mammalian growth, has long been overlooked as a simple nutrient. It has been revealed that aspartic acid serves as a key node molecule in the tricarboxylic acid cycle and the urea cycle, and possesses biological activities such as antioxidant, anti-inflammatory, immunomodulatory, and lipid metabolism-regulating effects, garnering widespread attention in the medical, food, and animal husbandry industries. Due to its chiral structure, aspartic acid naturally exists in two configurations, L- and D-forms, which exhibit certain differences in function and chemical properties. In animal husbandry, both configurations of aspartic acid have been applied in various animal models, where they have demonstrated roles in promoting growth, alleviating oxidative stress, modulating immune stress, and regulating gut microbial community structure. Therefore, aspartic acid holds promise as a conditionally functional amino acid for application in animal production. This review summarizes the physicochemical properties, metabolic pathways, and transport mechanisms of aspartic acid, outlines its in vitro synthesis methods, elucidates its physiological functions, and comprehensively reviews the current applications of its different configurations in the field of animal husbandry. The aim is to provide a theoretical foundation for the use of aspartic acid as a green, low-cost, and safe conditionally functional amino acid in animal husbandry.

## Simple Summary

Aspartic acid, a non-essential amino acid traditionally viewed as merely a protein building block, has emerged as a critical metabolic hub in the tricarboxylic acid and urea cycles, exhibiting antioxidant, anti-inflammatory, immunomodulatory, and lipid-regulating activities. It exists as L- and D-isomers with distinct functions: L-aspartic acid serves as a metabolic intermediate in energy and biosynthesis pathways, while D-aspartic acid acts as a signaling molecule via N-methyl-D-aspartate receptor-mediated neuroendocrine regulation. In livestock, both isomers enhance growth, alleviate oxidative stress, modulate immunity, and improve gut microbiota across multiple species. Under metabolic stress, endogenous aspartic acid may become insufficient, positioning it as a conditionally functional amino acid requiring supplementation. This review comprehensively examines aspartic acid’s physicochemical properties, metabolism, transport, in vitro synthesis, physiological roles, and applications in animal production, establishing a theoretical basis for its use as a sustainable, cost-effective, and safe functional amino acid in modern livestock systems.

## 1. Introduction

The livestock industry is facing unprecedented survival challenges. Heat stress caused by climate change has resulted in substantial annual economic losses for the global poultry sector, while heat stress in dairy cows leads to declining milk production [1]. The incidence of proliferative enteritis in pig farming has increased significantly in the post-antibiotic era, while traditional nutritional strategies demonstrate limited efficacy in regulating intestinal mucosal immunity [2]. Moreover, the livestock industry contributes to 9.2% of global greenhouse gas emissions [3]. These crises have exposed the limitations of classical nutrition science, where the protein–energy-centric paradigm struggles to address the intricate web of metabolic–immune–environment interactions.

These industry challenges underscore a fundamental gap in classical nutrition paradigms: the protein–energy-centric framework inadequately addresses the complex metabolic–immune–environment interactions that determine animal health and productivity under stress conditions. Functional amino acids have emerged as critical mediators that bridge this gap, not merely as building blocks for protein synthesis but as metabolic regulators and signaling molecules that modulate physiological responses to environmental challenges. Research shows that arginine regulates skeletal muscle fiber-type formation via the mammalian target of rapamycin (**mTOR**) signaling pathway [4], while glutamine maintains intestinal barrier integrity [5]. In this context, functional amino acids have evolved from mere nutrients into crucial mediators of metabolic regulation.

Aspartic acid, a non-essential amino acid in animal growth, has long been oversimplified as a structural unit in protein synthesis, while its significant value in animal husbandry has been severely underestimated. Research reveals that in energy metabolism hubs, aspartic acid’s regulatory role in the malate–aspartate shuttle—which regulates mitochondrial NADH/NAD^+^ balance—elevates adenosine triphosphate production in the liver [6]. In nitrogen cycling, aspartic acid serves as the essential amino group donor for argininosuccinate synthesis in the urea cycle, directly determining ammonia detoxification efficiency [7]. Notably, in neuroendocrine regulation, aspartic acid, as a N-methyl-D-aspartate receptor (**NMDAR**) agonist precursor, modulates the release of corticotropin-releasing hormone from the hypothalamus and reduces serum corticosterone levels in heat-stressed chicken models [8]. In immune metabolism, aspartic acid metabolism facilitates interleukin-1β (**IL-1β**) production in inflammatory macrophages [9]. From a microbiome perspective, aspartate improves ulcerative colitis via modulation of the receptor-interacting protein kinase 1 pathway and gut microbiota composition in mice [10].

However, a critical distinction must be made; while L-aspartic acid functions primarily as a metabolic intermediate participating in core biochemical pathways—the tricarboxylic acid cycle, urea cycle, and malate–aspartate shuttle—D-aspartic acid acts predominantly as a neuroendocrine regulator through its interaction with NMDAR [7,8]. This fundamental difference in mechanism has profound implications for aspartic acid’s respective applications in animal production, yet previous reviews have largely treated them under a unified “functional amino acid” concept without adequate distinction.

Therefore, it is essential to clarify the conceptual framework underlying this review. Under normal physiological conditions, aspartic acid is synthesized endogenously in sufficient quantities and is, therefore, classified as a non-essential amino acid. However, under specific stress conditions—including immune activation, oxidative stress, heat stress, and metabolic dysregulation—endogenous synthesis may be insufficient to meet elevated metabolic demands. In such contexts, aspartic acid functions as a conditionally functional amino acid, serving not only as a nutrient but also as a metabolic modulator and signaling molecule. This conditional framework provides the rationale for dietary supplementation and guides the distinction between nutritional support and metabolic intervention.

All in all, in the post-antibiotic era, extensive research focused on identifying substances with anti-inflammatory and antibacterial properties that are free from drug resistance and are environmentally friendly and safe, aiming to alleviate or resolve various issues in animal production. Functional amino acids have garnered significant attention from researchers due to their alignment with these criteria. Among them, aspartic acid, although not a nutrient requiring additional supplementation in mammals, exhibits metabolic mechanisms and biological activities that demonstrate its potential as a conditionally functional amino acid. It also shows broad application prospects in the field of animal husbandry. Therefore, this article reviews the physicochemical properties, metabolic and transport mechanisms in vivo, in vitro synthesis, biological activities, and current research status of aspartic acid in animal production. This lays a theoretical foundation for guiding further applications and mechanistic explorations of aspartic acid in the field of animal husbandry.

## 2. Physical and Chemical Properties

Aspartic acid is an acidic amino acid with the chemical formula C_4_H_7_NO_4_ and a molecular weight of 113.1. It appears as white crystals or powder, has a melting point of 300 °C, is slightly soluble in cold water, readily soluble in hot water, dilutes in acidic and alkaline solutions, is insoluble in ethanol or ether, and has a solubility of 5 g/L in water at 25 °C [11]. It is also combustible. Due to the presence of an asymmetric carbon atom in its structure, aspartic acid exhibits optical activity. It is dextrorotatory in acidic and aqueous solutions (L-aspartic acid) and levorotatory in alkaline solutions (D-aspartic acid) [12]. Naturally occurring aspartic acid in living organisms exists in both L- and D-configurations, but L-aspartic acid predominates in the body [12]. The enzymatic system for protein synthesis can only recognize and activate L-aspartic acid, attaching it to tRNA for subsequent protein synthesis on ribosomes [13]. Therefore, whether obtained from dietary sources or synthesized through metabolic pathways, aspartic acid must be in the L-form before entering the protein synthesis system. Additionally, D-aspartic acid does not participate in conventional protein synthesis in the body and is primarily produced through the racemization of L-aspartic acid. However, studies have revealed that trace amounts of D-aspartic acid from certain foods can also be absorbed by the small intestine and enter the body, laying the groundwork for further research on the regulation of D-aspartic acid [14]. Furthermore, research indicates that in metabolically inactive, long-lived proteins or tissues (such as the lens, white matter of the brain, and dentin), L-aspartic acid spontaneously racemizes with age, partially converting to D-aspartic acid. This process is considered a marker of tissue aging [15]. This stereospecificity extends beyond protein synthesis to receptor binding and signaling functions. D-aspartic acid exhibits high affinity for N-methyl-D-aspartate (**NMDA**) receptors, which are expressed in neural, endocrine, and reproductive tissues, enabling its role as a neurotransmitter and endocrine modulator. In contrast, L-aspartic acid has negligible affinity for NMDA receptors, explaining its divergent biological functions. This mechanistic distinction is fundamental to understanding their respective applications: L-aspartic acid primarily influences metabolism, antioxidant capacity, and immune function through its role as a metabolic intermediate, while D-aspartic acid exerts neuroendocrine effects through receptor-mediated signaling pathways.

## 3. Aspartic Acid Metabolism and Transporters

### 3.1. Aspartic Acid Metabolism

**Figure 1:**
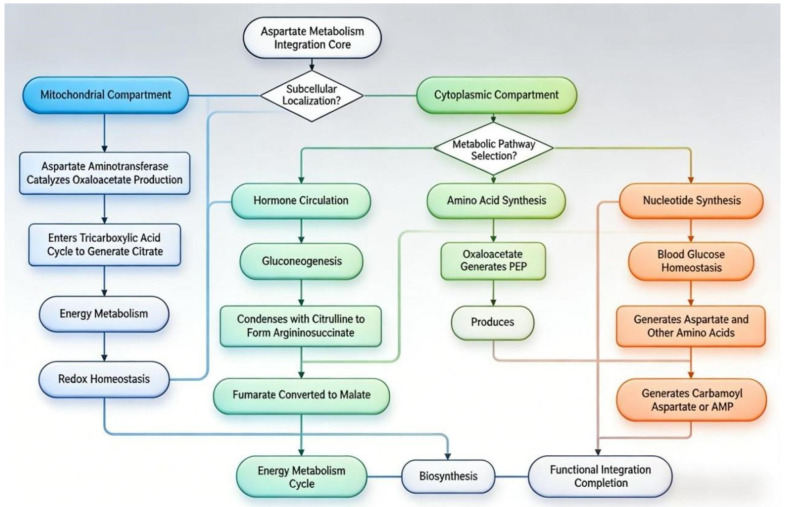
Aspartic acid as a central metabolic hub. In mitochondria, aspartate participates in the TCA cycle (via transamination to oxaloacetate) and the urea cycle (as a nitrogen donor for argininosuccinate). In the cytosol, it supports gluconeogenesis, nucleotide synthesis, and amino acid biosynthesis. Arrows indicate metabolic flux between compartments. Abbreviation: PEP = phosphoenolpyruvate.

Aspartic acid is a central nodal molecule that links protein metabolism, nucleic acid metabolism, and energy metabolism (Figure 1). In mammals, the synthesis and catabolism of aspartate primarily occur through transamination and the urea cycle [7]. Specifically, within the mitochondria of active tissues and organs (such as the liver, heart, and muscles), aspartate aminotransferase catalyzes the transfer of the amino group from aspartate to α-ketoglutarate, producing oxaloacetate and glutamate (transamination) [7]. This reaction not only connects amino acid metabolism with the tricarboxylic acid cycle but, more crucially, the generated oxaloacetate can serve as a gluconeogenic precursor. Via the phosphoenolpyruvate carboxykinase pathway, it is converted into glucose, which is vital for maintaining blood glucose stability during starvation or stress [16]. Simultaneously, aspartate is a direct nitrogen donor for the urea cycle. In the cytoplasm, catalyzed by argininosuccinate synthase, aspartate combines with citrulline, contributing its amino group to the urea molecule while itself being converted into fumarate. The fumarate can then replenish the tricarboxylic acid cycle [17]. This pathway is central to how aspartate processes and excretes excess nitrogen. With advancements in biochemistry, aspartate’s role as a key precursor in the synthesis of various macromolecules has been elucidated. In pyrimidine synthesis, aspartate condenses with carbamoyl phosphate to form carbamoyl aspartate, the starting point for all pyrimidine nucleotide synthesis [18]. In purine synthesis, aspartate acts as a nitrogen donor in the reactions converting inosine monophosphate to adenosine monophosphate and guanosine monophosphate [19]. Furthermore, aspartate is a direct biosynthetic precursor for amino acids such as asparagine, lysine, threonine, methionine, and isoleucine. Research has found that in cells with mitochondrial respiratory defects, aspartate synthesis dependent on the mitochondrial electron transport chain becomes a limiting factor for tumor cell proliferation [20]. This suggests that, beyond being a synthetic precursor, aspartate itself may function as a key metabolic signal. Additionally, due to the impermeability of the mitochondrial inner membrane to reduced nicotinamide adenine dinucleotide (**NADH**), shuttle systems are required to transfer reducing equivalents between the cytoplasm and mitochondria. Among these, the malate–aspartate shuttle is the primary system [21]. In this shuttle, the interconversion of aspartate and malate efficiently transports the reducing equivalents of cytoplasmic NADH into the mitochondrial matrix, where they enter the electron transport chain for oxidative ATP production. This process is not only essential for maintaining the redox balance (NAD^+^/NADH ratio) between the cytoplasm and mitochondria but also influences the rates of glycolysis and mitochondrial respiration [21].

It is worth noting that an important consideration for nutritional application is whether endogenous aspartic acid synthesis can become limiting under certain conditions. During immune activation, macrophages and lymphocytes exhibit increased demand for aspartate to support proliferation, cytokine production, and nucleotide synthesis. Studies in lipopolysaccharide-challenged piglets have demonstrated that endogenous aspartate synthesis may be insufficient to meet the elevated demands of hepatic ureagenesis, intestinal barrier maintenance, and immune cell activation [6,22]. Similarly, under oxidative stress, the malate–aspartate shuttle’s demand for aspartate increases to maintain NAD^+^/NADH balance and support glutathione regeneration [7]. These observations suggest that while aspartic acid is non-essential under basal conditions, it may become conditionally essential during metabolic stress, providing the physiological rationale for dietary supplementation.

### 3.2. Aspartic Acid Transporters

**Figure 2:**
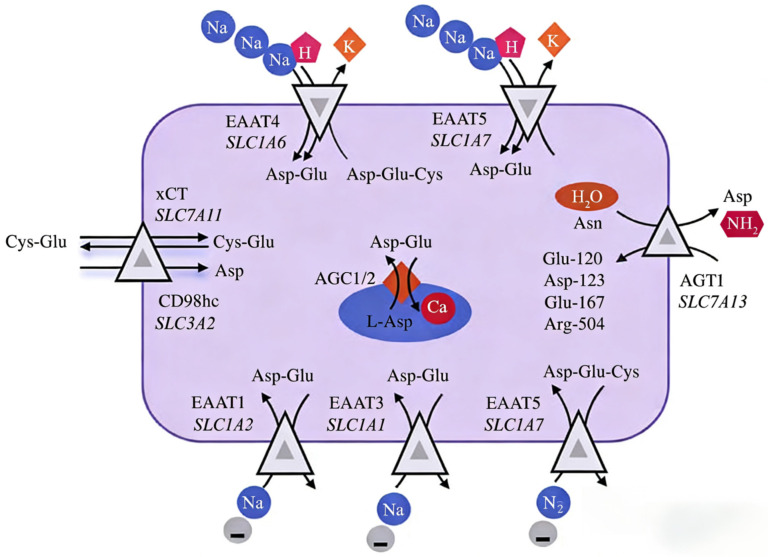
Aspartic acid transport systems. EAAT family transporters (SLC1A1, SLC1A3, and SLC1A7) mediate Na^+^-dependent Asp/Glu uptake. xCT (SLC7A11) exchanges extracellular Cys for intracellular Glu/Asp. Mitochondrial AGC transporters (SLC25A12/13) exchange aspartate for glutamate, resulting in the malate–aspartate shuttle. Ion dependencies and key residues are indicated. Abbreviation: Asp = aspartic acid; Glu = glutamate; Arg = arginine; Cys = cystine.

Aspartic acid transporters are present on the plasma membrane and organelle membranes of mammalian cells, forming channels for aspartate shuttling across cells. Aspartate transporters mainly include acidic aspartate transporters, neutral aspartate transporters, and aspartate transporters located on the mitochondrial membrane (Figure 2). Acidic aspartic acid transporters primarily consist of the EAAT protein family, the xCT amino acid transporter, and the AGT1 amino acid transporter. The EAAT protein family is encoded by the genes *SLC1A3* (EAAT1), *SLC1A2* (EAAT2), *SLC1A1* (EAAT3), *SLC1A6* (EAAT4), and *SLC1A7* (EAAT5) [23]. Studies have revealed that, except for the EAAT3 transporter, which transports glutamate, aspartate, and cysteine, the other EAATs transport both glutamate and aspartate, and they exhibit tissue specificity across various organs and cell types [23]. The xCT amino acid transporter is a sodium-independent transporter encoded by the *SLC7A11* gene, responsible for transporting aspartate, cysteine, and glutamate [24]. The AGT1 amino acid transporter, encoded by the *SLC7A13* gene, can transport glutamate and aspartate, and in mammals, AGT1 catalyzes the transamination of asparagine, promoting glyoxylate metabolism [25]. Neutral aspartate transporters mainly include the SNAT8 and SNAT10 amino acid transporters. The SNAT8 transporter, encoded by the *SLC38A8* gene, not only transports aspartate but is also a specific glutamine transporter [26]. The SNAT10 amino acid transporter, encoded by the *SLC38A10* gene, is specifically localized in secretory organelles, expressed in both excitatory and inhibitory neurons in the mouse brain, primarily in the endoplasmic reticulum and Golgi apparatus. A study using primary cortical cells from *SLC38A10* gene knockout mice has shown that the SNAT10 protein is involved in glutamate-sensing signaling pathways that regulate glutamate homeostasis and mTOR signal transduction [27]. The aspartate transporter on the mitochondrial membrane is primarily the aspartate–glutamate carrier (**AGC**), which has two isoforms identified as AGC1 (encoded by the *SLC25A12* gene) and AGC2 (encoded by the *SLC25A13* gene) [28]. The AGC1 is expressed in the heart, skeletal muscle, and brain, while AGC2 is expressed in the liver and gastrointestinal tract. The functions of both AGC1 and AGC2 are regulated by calcium ions.

## 4. In Vitro Synthesis of Aspartic Acid

In vitro synthesis methods for aspartic acid mainly include chemical synthesis and microbial synthesis. Early research on aspartic acid synthesis primarily relied on chemical methods, using maleic anhydride or fumaric acid as raw materials, and proceeding through multi-step reactions such as amination and hydrolysis. Although this method offers a clear reaction pathway, it requires harsh conditions (high temperature and pressure), involves toxic catalysts, yields low optical purity (predominantly DL-racemic mixtures), and causes significant environmental pollution. To obtain L-aspartic acid, additional chiral separation is necessary, leading to a substantial increase in costs. With advancements in biocatalytic technology, routes combining chemical synthesis and enzymatic catalysis have become a research focus. The representative fumarate–ammonia–aspartase system has been widely adopted. Chemically synthesized fumaric acid reacts with ammonia under the catalysis of aspartase (derived from microorganisms such as *Escherichia coli*), efficiently and specifically producing L-aspartic acid [29]. This approach combines the raw material advantages of chemical methods with the stereoselectivity of enzymatic catalysis, achieving a conversion rate of over 99% and an optical purity exceeding 99.5%. It has become one of the mainstream industrial production processes. Early microbial methods mainly depended on wild-type strains such as Corynebacterium glutamicum for fermentation production, with efforts focused on optimizing culture media and fermentation conditions to improve yield. However, wild-type strains face challenges such as complex metabolic pathways, numerous by-products, and low productivity, limiting their industrial application [30]. With the development of synthetic biology, researchers have enhanced the aspartic acid synthesis pathway by strengthening key steps, increasing the accumulation of precursors like oxaloacetate, knocking out or downregulating key genes in competing pathways, and optimizing aspartic acid efflux systems [31]. Representative engineered strains have achieved yields of about 100 g/L, utilizing renewable carbon sources as substrates, thereby reducing environmental pollution [31,32]. Future research could focus on developing synthesis routes utilizing non-food biomass (e.g., cellulose and algae) as raw materials to reduce dependence on food-based carbon sources [32]. Additionally, exploring the use of one-carbon feedstocks such as CO_2_ for aspartic acid synthesis through coupled chemical–biological carbon fixation could enable carbon-negative manufacturing [32,33].

From an animal nutrition perspective, the production method has important implications for feed applications. Chemical synthesis typically yields racemic mixtures requiring separation, while enzymatic and microbial methods can produce stereospecific L- or D-forms with high purity. Feed-grade aspartic acid must meet appropriate purity standards and be free from toxic intermediates or catalysts. The cost-effectiveness of production methods directly influences economic viability for livestock applications, with current microbial fermentation approaches showing promise for large-scale, sustainable production [29,31,32].

## 5. Biologic Activity of Aspartic Acid

The antioxidant, immune-regulatory, and metabolic functions of aspartic acid described in this section are supported by evidence from diverse experimental systems. It is important to distinguish between mechanistic evidence derived from in vitro systems and rodent models and the validated physiological outcomes observed in livestock species. While cell culture studies provide valuable insights into molecular mechanisms, their direct extrapolation to production animals requires careful consideration of species-specific metabolism, dosage, and physiological context. Where possible, this review prioritizes evidence from target livestock species and explicitly notes when conclusions rely on non-livestock models.

### 5.1. Relieve Oxidative Stress

Aspartic acid itself is not a classic antioxidant, but through its metabolites and participation in biochemical networks, it plays a multi-level and indirect antioxidant function in the body. Aspartic acid has an antioxidant function through three core pathways. (1) Maintaining glutathione and glutamine synthesis: aspartic acid undergoes transamination to form oxaloacetic acid, which enters the TCA cycle to produce α-ketoglutaric acid (**α-KG**), which is a precursor of glutamate [34,35]. Glutamate serves as an essential substrate for synthesizing glutamine and glutathione [36]. Research has demonstrated that L-aspartic acid exhibits hepatoprotective effects in non-alcoholic fatty liver disease. Mechanistically, this explains how L-aspartic acid is metabolized into L-glutamine, L-arginine, and glutathione metabolites that prevent hepatic lipid peroxidation and enhance antioxidant capacity in the liver [37]. (2) Participation in carnosine synthesis: aspartic acid undergoes decarboxylation to form β-alanine, which is then converted into carnosine. This compound directly scavenges hydroxyl radicals and peroxynitrite. Hoffman [38] highlighted that β-alanine constitutes a crucial component of carnosine. Supplementing with β-alanine enhances muscle carnosine levels, thereby delaying fatigue during high-intensity exercise. Elevated carnosine concentrations may improve cognitive function and stress resilience; these benefits are attributed to carnosine’s potential antioxidant effects. (3) Regulating intestinal microorganisms to produce antioxidants: it was found that during oxidative stress, aspartic acid reduced the abundance of *Megasphaera* and increased the abundance of *Fusobacteriaceae*, which depended on aspartic acid metabolism, regulating the enhancement of antioxidant eicosapentaenoic acid production mediated by the microbiota. From a mechanistic perspective, the application of exogenous aspartic acid regulates antioxidant responses in intestinal epithelial cells by modulating the RIP3/MLKL and RIP1/Nrf2/NF-κB signaling pathways [34]. This mechanism helps eliminate excessive reactive oxygen species while maintaining mitochondrial function and cellular viability. These mechanisms have been primarily elucidated using in vitro and rodent models. In livestock models, a study showed that adding 1% aspartic acid to weaned piglet diets can enhance average daily weight gain and feed intake while reducing serum MDA and ROS levels [39]. However, the antioxidant properties of aspartic acid have limitations: excessive amounts may convert to glutamic acid, which overactivates NMDA receptors and induces excitotoxic oxidative stress. In the future, through dose screening and precise regulation, the metabolic network of aspartic acid is expected to become an important potential target for antioxidant therapy and animal health management. Despite these promising effects, several translational limitations warrant consideration. First, the doses demonstrating antioxidant effects in livestock studies are substantially higher than normal physiological exposure levels, suggesting that they represent metabolic interventions rather than nutritional supplementation per se. Second, the relationship between aspartic acid supplementation and oxidative stress follows a biphasic pattern: while moderate supplementation enhances antioxidant capacity, excessive amounts may be converted to glutamate, potentially overactivating NMDA receptors and inducing excitotoxic oxidative stress in neural tissues. Third, the long-term safety of chronic high-dose supplementation has not been systematically evaluated in production animals, particularly regarding potential impacts on amino acid balance, nitrogen excretion, and metabolic load on hepatic and renal function.

### 5.2. Regulate Immune Function

The immune function of the body protects the host from environmental stimulation and maintains the physiological stability of the host through immune cells [40]. Once immune cells sense disease or other damage, they are rapidly activated to perform the corresponding immune mechanism [41]. Immune cells in the body mainly include T-cells, B-cells, and macrophages. Aspartic acid can regulate the function of T-cells and the polarization of macrophages. In-depth research on the regulation of T-cell function has mainly been conducted at the cellular and rodent levels. It has been reported that a lack of aspartic acid in mitochondria forces the endoplasmic reticulum of T-cells to produce transmembrane tumor necrosis factor (**TNF**), which leads to synovitis [42]. Wu [43] demonstrated that mitochondrial aspartic acid deficiency disrupts the regeneration of NADH as a metabolic cofactor. This leads to the ADP-ribosylation of the endoplasmic reticulum (**ER**) sensor GRP78/BiP, resulting in membrane expansion of ribosome-rich ER. The process promotes co-translational translocation and enhances transmembrane TNF biogenesis. Conversely, supplementation with exogenous aspartic acid enables ER membrane expansion while inhibiting TNF release and rheumatoid tissue inflammation. The findings demonstrate that aspartic acid regulates T-cell function to suppress the secretion of inflammatory cytokine TNF [43]. Notably, Wang [9] discovered that aspartic acid promotes the secretion of IL-1β in M1 macrophages. Mechanistically, aspartic acid activates hypoxia-inducible factor-1α (**HIF-1α**) and inflammasomes (**NLRP3**) while increasing asparagine levels in its metabolic pathway. Aspartic acid further accelerates the activation of HIF-1α and NLRP3 signaling pathways, thereby enhancing macrophage production of inflammatory cytokine IL-1β. Additionally, aspartic acid supplementation (administering 0.5 mg/mL of aspartic acid in drinking water for mice) enhances macrophage-mediated inflammatory responses, which exhibit bactericidal effects in the intestinal tracts of the mice [9]. In addition, aspartic acid may enable M2 activation by promoting the TCA cycle and producing purine metabolites [44,45]. However, this study found that aminooxyacetic acid (an inhibitor of AST) inhibited aspartic acid synthesis, hindered the pro-inflammatory phenotype of M1 macrophages, and promoted the anti-inflammatory M2 phenotype, indicating that aspartic acid may impair M2 polarization [46]. Therefore, the role of aspartic acid in M2 polarization remains controversial. In conclusion, given the potential functions of aspartic acid and its metabolites in T-cells and macrophages, it is feasible to regulate immune cell function by targeting aspartic acid metabolism to modulate immune capacity, thereby preventing or treating microbial infections and other animal diseases. The role of aspartic acid in macrophage polarization requires nuanced interpretation due to apparently conflicting evidence. Studies using aminooxyacetic acid (an inhibitor of AST) have shown that inhibiting aspartate metabolism promotes the anti-inflammatory M2 phenotype while suppressing the pro-inflammatory M1 phenotype [44,45,46]. This suggests that endogenous aspartate metabolism may favor M1 polarization. However, other studies have demonstrated that aspartate-derived metabolites support M2 activation through TCA cycle intermediates and purine metabolites. These apparently contradictory findings may reflect context-dependent effects: under inflammatory conditions, aspartate may fuel the enhanced metabolic demands of M1 macrophages, while under homeostatic conditions, it may support the tissue-repair functions of M2 macrophages. This metabolic flexibility highlights the complexity of translating mechanistic findings from isolated cell systems to whole-animal physiology. However, these mechanisms are primarily based on studies involving in vitro cell models and rodent research, and their applicability in livestock production still requires further investigation. Notably, the study by Wang [9] further demonstrated that exogenous supplementation with 0.05% aspartic acid regulates the secretion of f IL-1β by macrophages in the piglet intestine, exhibiting significant bactericidal effects. This suggests the potential of aspartic acid for immune modulation in livestock production.

### 5.3. Regulation of Glucose Metabolism

Aspartic acid is a glucogenic amino acid that, in addition to promoting sugar synthesis, is closely related to insulin resistance. Insulin resistance is primarily characterized by reduced tissue sensitivity to insulin and decreased glucose utilization, leading to elevated blood glucose levels [47]. A study by Vangipurapu [48] found that aspartic acid is one of the amino acids predictive of type 2 diabetes models, and tyrosine, alanine, isoleucine, aspartic acid, and glutamic acid are associated with reduced insulin secretion. Subsequently, Takahashi [14] reported that supplementing aspartic acid in ruminant diets reduces insulin secretion without affecting blood glucose levels, though the specific mechanism of action still requires further research. After animals consume feed, blood glucose levels rise, and insulin secreted by pancreatic β-cells binds to insulin receptors on target cell membranes, activating the downstream phosphatidylinositol-3-kinase (**PI3K**)/protein kinase B (**Akt**) signaling pathway [49]. Activation of PI3K/Akt can also mediate glucose transporter 4 (**GLUT4**) in muscle and fat cells to enhance glucose utilization [47]. Huang [50] found that glutamine can activate the expression of proteins related to the PI3K/Akt signaling pathway, promote sugar synthesis, inhibit gluconeogenesis, improve insulin resistance in obese mice, and reduce blood glucose levels. Therefore, glutamine has the potential to regulate the PI3K/Akt signaling pathway to ameliorate insulin resistance. Glutamine is a precursor for the synthesis of aspartic acid. It has been reported that aspartic acid can activate NMDAR, thereby influencing the downstream PI3K/Akt signaling pathway to promote spermatogonial proliferation [51]. In summary, aspartic acid promotes sugar synthesis, serves as one of the predictive amino acids for type 2 diabetes, and reduces insulin secretion without significantly affecting blood glucose levels. The specific mechanisms involved still require further investigation. Future research could explore the effects of aspartic acid on NMDAR and the PI3K/Akt signaling pathway, as well as related proteins, to further elucidate the role of aspartic acid in glucose metabolism and the mechanisms of insulin resistance. It is worth noting that the relationship between aspartic acid and glucose metabolism illustrates the critical distinction between mechanistic insight and practical application. While epidemiological studies have identified aspartic acid as one of nine amino acids predictive of type 2 diabetes risk in humans, this association does not imply causation and may reflect broader metabolic dysregulation rather than a direct effect of aspartic acid per se. In livestock, the observation that aspartic acid supplementation reduces insulin secretion without affecting blood glucose levels suggests a potential role in modulating insulin sensitivity, but the underlying mechanisms—possibly involving NMDAR activation in pancreatic islets or alterations in gut hormone secretion—remain poorly characterized. Future research should prioritize elucidating these mechanisms in target livestock species rather than relying on extrapolation from rodent or human studies.

### 5.4. Regulation of Fat Metabolism

Early studies revealed a correlation between aspartic acid consumption and increased lipid synthesis in proliferating cells. Preliminary experiments using in vitro hepatocellular carcinoma cell models showed that inhibiting aspartate aminotransferase reduced the cytoplasmic NADH/NAD^+^ ratio, indirectly affecting fatty acid synthase activity [52]. These early studies proposed a link between aspartate and lipid metabolism, though the mechanism remained unclear. Subsequently, the aspartate–malate shuttle was identified as directly providing essential reducing power in the form of β-nicotinamide adenine dinucleotide phosphate (**NADPH**) for fatty acid synthesis [53]. A series of cell model studies (HeLa, HEK293, liver cancer cell lines) using isotope-labeled metabolic flux analysis confirmed that approximately 30% of the NADPH required for fatty acid synthesis is derived from this shuttle pathway [54,55]. Further validation through gene silencing experiments demonstrated that knocking down malic enzyme 1 led to a decreased NADPH/NADP^+^ ratio and reduced lipid synthesis [54]. These findings established a direct causal relationship between aspartate metabolism and lipid synthesis. In addition, in vivo studies in mice confirmed the physiological relevance of aspartate in regulating lipid metabolism. Mice with liver-specific knockout of aspartate aminotransferase exhibited a decreased hepatic triglyceride content, elevated plasma free fatty acids, and a significant reduction in high-fat diet-induced fatty liver [56]. These phenotypes were consistent with diminished NADPH production and restricted fatty acid synthesis. Research has uncovered a more direct role for aspartate. In a 3T3-L1 preadipocyte differentiation model, under aspartate-limited conditions, the expression of adipogenic markers peroxisome proliferator-activated receptor γ was downregulated, and lipid droplet formation decreased [57]. This indicates that aspartate not only provides biosynthetic precursors but also participates in the transcriptional regulation of adipogenesis. In summary, aspartate influences lipid metabolism by supplying NADPH reducing power for fatty acid synthesis, maintaining TCA cycle integrity, serving as a precursor for acetyl-CoA replenishment pathways, and participating in the transcriptional regulation of adipogenesis. These mechanistic insights have largely been gained from tumor cell lines and knockout mouse models, providing a molecular basis for understanding aspartic acid’s function. Future research should further clarify the tissue-specific regulatory networks of aspartate metabolism (e.g., in liver and adipose tissue) and the impact of microenvironmental aspartate fluctuations on systemic lipid homeostasis. In addition, an important consideration for practical application is the tissue-specific nature of aspartate’s effects on lipid metabolism. In the liver, aspartate-derived NADPH supports de novo lipogenesis, potentially contributing to hepatic lipid accumulation under conditions of excess energy intake. Conversely, in adipose tissue, aspartate may support adipocyte differentiation and function. This tissue-specific duality has implications for supplementation strategies: while enhanced lipogenesis may be undesirable in finishing pigs where excessive fat deposition reduces carcass quality, it may be beneficial in early growth stages where adipose tissue development supports energy storage and insulation. Future research should, therefore, consider not only the magnitude of effects but also their tissue distribution and the resulting impact on whole-body metabolic homeostasis.

## 6. Application of Aspartic Acid in Animal Production

Current research in the animal production field primarily focuses on the practical application effects of L-aspartic acid and D-aspartic acid in various stress models (Table 1). It is particularly important to note that many of the studies listed in Table 1, especially those in pigs and poultry, used supplementation levels (e.g., 0.5–2% of the diet) that are considerably higher than normal physiological requirements. This level of supplementation should be understood as a “metabolic intervention” rather than traditional “nutritional supplementation.” The rationale is to meet a sharply increased metabolic demand during specific stress or physiological phases, or to activate specific signaling pathways through exogenous supply. While the efficacy of this intervention strategy has been confirmed in several studies, its long-term safety—for instance, the potential impact on amino acid balance, nitrogen excretion, and the metabolic load on the liver and kidneys—still requires systematic evaluation in future research. Specifically, in conventional animal model trials with growing piglets, supplementation with 1% L-aspartic acid and 1% D-aspartic acid both improved growth performance and microbial diversity in piglets. However, L-aspartic acid increased the abundance of Actinobacteria and Bacteroidetes while reducing the relative abundance of Firmicutes. In contrast, D-aspartic acid increased the relative abundance of *Clostridium sensu stricto 1* and Enterobacteriaceae [58]. This suggests that under identical rearing conditions, the two configurations of aspartic acid have slightly different effects on the gut microbiota of piglets, although phenotypic outcomes are consistent. The underlying reasons require further investigation. It is worth noting that the interpretation of microbiota changes requires caution. While increased microbial diversity is generally considered beneficial, specific taxonomic shifts must be evaluated in a physiological context. For example, the increase in Enterobacteriaceae abundance observed with D-aspartic acid supplementation, while presented as a phenotypic change, may not necessarily indicate improved gut health. Enterobacteriaceae include potentially pathogenic species, and their expansion could reflect dysbiosis or increased inflammatory potential. Similarly, changes in Firmicutes/Bacteroidetes ratios, while commonly interpreted in relation to obesity and metabolism, have variable implications depending on the specific species involved and the host’s physiological state. Future studies should, therefore, move beyond 16S rRNA-based taxonomic descriptions to functional metagenomic analyses that assess the actual metabolic capacity of the altered microbiota.

Furthermore, in an oxidative stress model induced by hydrogen peroxide in weaned piglets, the addition of 1% L-aspartic acid to the basal diet mitigated the reduction in feed intake caused by oxidative stress, improved feed conversion efficiency, and enhanced serum antioxidant enzyme activity [59]. Similarly, in an oxidative stress model in boars, supplementation with 2% L-aspartic acid reduced serum malondialdehyde concentration [59]. To date, no studies have been found on the effects of D-aspartic acid in alleviating oxidative stress in animal production. Research by Venditti [60] indicated that D-aspartic acid has a mitigating effect on oxidative stress in rat testicular tissue induced by cadmium, although the specific mechanism remains unknown. Therefore, L-aspartic acid holds application prospects in alleviating oxidative stress in animals, while D-aspartic acid shows potential in this regard and may have targeted therapeutic effects on oxidative stress in reproductive organs. It has been reported that infection and inflammation can lead to muscle atrophy [61]. Wang [61] found that in a stress model induced by lipopolysaccharide in weaned piglets, supplementation with 0.5% and 1% L-aspartic acid prevented muscle atrophy by inhibiting the phosphorylation of AMPKα and FOXO1. Li [58] discovered that in an oxidative stress model in boars, adding 2% L-aspartic acid increased transforming growth factor-β1 in the epididymis and testes, exerting anti-inflammatory effects. Similarly, it is noteworthy that in a normal piglet model, D-aspartic acid alleviated jejunal inflammation mediated by the genes TLR4, NOD1, and MyD88 [58]. Thus, both configurations of aspartic acid can regulate inflammation in piglets at the transcriptional level, but their mechanisms of action differ, and their specific effects have not been comparatively analyzed in research. In addition, the dosage levels employed in the cited studies warrant careful interpretation. At these elevated levels, aspartic acid supplementation likely functions as a metabolic intervention rather than nutritional supplementation per se. Several safety considerations arise from this observation: First, high-dose supplementation may disrupt the delicate balance of dietary amino acids, potentially inducing antagonisms with other dicarboxylic amino acids or affecting branched-chain amino acid metabolism. Second, the metabolic load imposed by excess aspartate on hepatic transamination and ureagenesis may not be sustainable during chronic administration. Third, the potential for excitotoxic effects mediated by excessive glutamate production (from aspartate transamination) warrants investigation, particularly in neural tissues. Future research should establish dose–response relationships and identify safety thresholds before widespread commercial application can be recommended.

**Table 1:** The application effects of L- and D-aspartic acid in animal production.

Items	Optimum Dosages	Varieties	Models	Phenotypic Changes	References
L-aspartic acid	Feed (1%)	Piglet	Normal model	Reduced feed intake and inflammation. Increased daily weight gain. Regulated gut microbiota.	[58]
	Feed (0.5% or 1%)	Piglet	LPS-induced liver injury model	Relieved liver damage and inflammation. Improved liver energy metabolism.	[22]
	Feed (0.5% or 1%)	Piglet	LPS-induced intestinal injury model	Improved intestinal injury status and intestinal energy metabolism.	[6]
	Feed (1%)	Piglet	Oxidative stress model	Improved feed conversion ratio and relieved oxidative stress.	[39]
	Feed (5.7%)	Broiler	Low-protein feed model	No significant effect on growth performance.	[62,63]
	Feed (5.7%)	Layer	Low-protein feed model	No significant effect on egg production ratio.	[62,63]
D-aspartic acid	Feed (1%)	Piglet	Normal model	Reduced feed intake and inflammation. Increased daily weight gain. Regulated gut microbiota.	[58]
	Subcutaneous injection (44 mg/kg)	Sheep	Normal model	Increased luteinizing hormone.	[64]
	Feed (0.5%)	Piglet	Low-protein feed model	Improved meat quality and increased back fat. Regulated gut microbiota.	[56]
	Oral administration (15 mmol/kg)	Broiler	Heat stress model	Reduced rectal temperature. Alleviated heat stress.	[8]
	Feed (200 mg/kg)	Broiler roosters	50-week-old model or 55-week-old model	Increased testosterone, fertility ratio, sperm motility, and post-thaw sperm motility. Induced weight loss.	[65,66]

Abbreviation: LPS = lipopolysaccharide.

A growing body of research indicates that non-essential amino acids can stimulate feed intake and growth rates in animals. In conventional feeding models, slow-growing broilers show a preference for diets with low essential amino acids (lysine, methionine, and threonine) and high non-essential amino acids (alanine, aspartate, and asparagine) in two-choice preference tests compared to fast-growing broilers [67]. This suggests that slow-growing broilers tend to prefer diets with a higher proportion of non-essential amino acids. Studies have found that supplementing L-aspartic acid in low-protein diet models has no significant effect on the growth performance of fast-growing broilers or the egg production rate of laying hens [62,63]. However, in low-protein diet models for piglets, supplementation with D-aspartic acid improves growth performance, meat quality, and increases backfat deposition. It is worth noting that low-protein diets often lead to excessive fat deposition in animals, which limits their application during the piglet growth stage [56]. In the finishing stage, however, pigs exhibit slower growth rates, stable muscle tissue development, and a gradual increase in the proportion of adipose tissue. This stage requires reducing dietary protein content and increasing energy supply to shorten the finishing period, but a drawback is the adverse effect on meat quality at slaughter. Therefore, supplementation with D-aspartic acid during the finishing stage of pigs may be more beneficial for improving pork quality and shortening the finishing cycle. However, this conclusion still requires further validation through experimental research.

Heat stress poses a significant challenge to the poultry industry. Due to the absence of sweat glands and full feather coverage, poultry are highly susceptible to heat stress under high-temperature conditions, leading to reduced feed intake, feed conversion efficiency, egg production rate, and immune function, thereby causing substantial economic losses [63]. A study on sheep revealed that intracerebroventricular injection of 100 nmol/kg or 200 nmol/kg of L-aspartic acid increased heat production, reduced heat loss, and elevated rectal temperature, indicating that L-aspartic acid metabolism can serve as a target for thermoregulation and promote heat production [63,68]. However, a study by Erwan [8] found that oral administration of 15 mmol/kg D-aspartic acid reduced mitochondrial heat production, lowered rectal temperature in poultry, alleviated heat stress, and improved production performance, whereas L-aspartic acid did not mitigate heat stress in poultry. These findings suggest that L-aspartic acid promotes heat production in animals, while D-aspartic acid can alleviate heat stress in animals. The contrasting effects of L- and D-aspartic acid on thermoregulation illustrate the fundamental distinction between their mechanisms of action. L-aspartic acid, as a metabolic intermediate, supports oxidative phosphorylation and ATP production, which may increase metabolic heat production as a byproduct of enhanced energy metabolism. In contrast, D-aspartic acid’s effects are mediated through NMDAR signaling in the hypothalamus, where it modulates the release of the corticotropin-releasing hormone and alters the central set-point for thermoregulation [59,69]. This mechanistic distinction explains why D-aspartic acid, but not L-aspartic acid, effectively alleviates heat stress in poultry—it targets the central neuroendocrine pathways that coordinate stress response rather than peripheral metabolism. This example underscores the importance of matching the specific isomer to the targeted physiological outcome. This example underscores the importance of matching the specific isomer to the targeted physiological outcome: the L-form primarily influences peripheral energy metabolism, which may be accompanied by increased heat production, whereas the D-form modulates the stress response via central neuroendocrine pathways, demonstrating unique value in alleviating heat stress in poultry.

Currently, the assessment of animal reproductive performance primarily relies on indicators such as hormone levels, sperm and oocyte development and maturation, as well as postpartum litter size, the number of healthy offspring, and hatchability [70]. Common hormones used to evaluate reproductive performance include the luteinizing hormone, follicle-stimulating hormone, estrogen, progesterone, prostaglandins, and testosterone. Testosterone, a steroid hormone derived from Leydig cells, is crucial for maintaining male libido and promoting the development of secondary sexual characteristics [65]. In this study, we found that supplementing the basal diet of 50- and 55-week-old breeder roosters with 200 mg/kg D-aspartic acid increased circulating testosterone levels, sperm motility, and sperm concentration, thereby improving fertility and hatchability [65]. Mechanistically, D-aspartic acid upregulates the expression of the STAR gene, which promotes the transfer of cholesterol from the cytoplasm to the mitochondria in Leydig cells [66]. Within the mitochondria, cholesterol is converted to pregnenolone by P450scc, and pregnenolone serves as the precursor for all steroid hormones [66]. Elevated testosterone levels are directly correlated with an increased proportion of motile sperm. Therefore, the improved reproductive capacity of aging roosters is associated with D-aspartic acid’s upregulation of STAR gene expression, which enhances circulating testosterone levels, sperm motility, and concentration. Sperm motility, concentration, and morphology, as well as follicle size, number, and morphology, are key indicators reflecting animal reproductive quality [65]. Research has revealed that D-aspartic acid can protect bull sperm motility and concentration, enhancing their ability to support embryonic development, likely due to its antioxidant capacity [71]. Research on D-aspartic acid has predominantly focused on male reproductive capacity, with relatively limited studies in females [72]. Currently, only one study indicates that D-aspartic acid can also promote oocyte development in female lizards [73]. Notably, no study to date has demonstrated a direct impact of L-aspartic acid on animal reproductive performance. L-aspartic acid and D-aspartic acid are chiral molecules, sharing the same molecular formula but possessing stereospecific three-dimensional structures. Research has found that D-aspartic acid binds to NMDAR in the brain and reproductive system, serving as a key signal for stimulating hormone secretion. In contrast, L-aspartic acid has very low affinity for NMDA receptors, which may explain its less pronounced effects on animal reproductive capacity compared to D-aspartic acid [59,69]. In summary, D-aspartic acid promotes testicular development, improves sperm quality, and enhances testosterone levels in male animals. Compared to L-aspartic acid, D-aspartic acid is more suitable for regulating animal reproductive performance, likely due to their differing affinities for NMDAR. While the effects of D-aspartic acid on male reproduction are well-documented in rodents and poultry, several limitations should be acknowledged. First, most studies have been conducted in roosters and rodents, with limited validation in mammals of commercial importance, such as boars, bulls, or rams. Second, the optimal dosage, timing, and duration of supplementation for reproductive enhancement have not been systematically established across species. Third, the potential effects on female reproduction remain largely unexplored, with only one study in lizards suggesting possible benefits for oocyte development. Fourth, the mechanism of action—upregulation of STAR gene expression and enhancement of cholesterol transport into mitochondria—suggests that D-aspartic acid may be most effective in animals with suboptimal steroidogenesis, such as aged breeders, but may have limited effects in young, reproductively healthy animals. These caveats should guide future research priorities and temper expectations for universal application.

## 7. Conclusions

This review examines aspartic acid’s physiological functions and applications in animal production, emphasizing the distinct roles of its stereoisomers. Three key conclusions emerge: First, L-aspartic acid and D-aspartic acid should be considered separately. L-aspartic acid functions primarily as a metabolic intermediate in energy metabolism and biosynthesis, while D-aspartic acid acts as a signaling molecule through NMDA receptor pathways, particularly affecting reproduction and thermoregulation. Second, aspartic acid is conditionally essential during metabolic stress. Although endogenous synthesis suffices under normal conditions, supplementation becomes beneficial during immune activation, oxidative stress, or metabolic dysregulation when demand exceeds supply. Third, significant knowledge gaps remain. Mechanisms derived from in vitro and rodent studies require validation in livestock species under commercial conditions. Safety considerations are inadequately addressed, with most studies using high doses (0.5–2% of diet) that represent metabolic interventions rather than nutritional supplementation. Long-term effects on amino acid balance, nitrogen excretion, and organ load need systematic evaluation. Practical applications must consider sustainable production contexts. L-aspartic acid shows promise for alleviating oxidative stress, while D-aspartic acid demonstrates potential for improving reproductive performance in aged breeders and mitigating heat stress in poultry. However, benefits must be weighed against costs and environmental impact. Future research priorities include elucidating tissue-specific metabolic networks in livestock, establishing dose–response relationships and safety thresholds, developing stage-specific supplementation strategies, investigating synergies with other additives, validating reproductive benefits in mammals, and integrating microbial synthetic biology for cost-effective production. Addressing these priorities will enable aspartic acid’s transition from a research compound to a practical tool in post-antibiotic livestock production.

## Data Availability

No new data were created or analyzed in this study. Data sharing is not applicable.
